# Acute compartment syndrome in children; beware of “silent” compartment syndrome: A CARE-compliant case report

**DOI:** 10.1097/MD.0000000000020504

**Published:** 2020-06-05

**Authors:** Benjamin Frei, Vivienne Sommer-Joergensen, Stefan Holland-Cunz, Johannes Mayr

**Affiliations:** Department of Pediatric Surgery, University Childrenʼs Hospital Basel (UKBB), Basel, Switzerland.

**Keywords:** children, compartment syndrome, fasciotomy, hand, intrinsic minus position, silent

## Abstract

**Rationale:**

Acute compartment syndrome (ACS) is a feared complication following traumatic injuries. We describe the occurrence of silent ACS of the hand in a 2-year old patient with atypical symptoms.

**Patient concerns:**

Our patient experienced massive swelling but minimal pain of the hand after a heavy bistro table with a stone tabletop had fallen on the right hand.

**Diagnosis:**

After monitoring the development of ACS for 1 night, we noted increased firmness of the swelling and impaired perfusion of the skin covering the palm and dorsum of the hand. Notably, the patient held the hand in an intrinsic minus position but did not complain of pain after administration of only a single (weight-matched) dose of ibuprofen. Our suspicion of ACS was confirmed intraoperatively because of the elevated intramuscular pressure (up to 60 mm Hg) in several hand compartments.

**Interventions:**

We performed surgical fasciotomy of all hand compartments, followed by temporary coverage of the wounds with Epigard synthetic skin substitute. The wounds were closed stepwise after 2 and 7 days, and occupational therapy was initiated after 3 weeks.

**Outcomes:**

At the 1-year follow-up, we noted unrestricted wrist and finger functions of the patient. The parents reported that there was no difference in the use of the 2 hands during daily activities.

**Lessons:**

The possible development of ACS should be borne in mind even in the absence of marked pain. Although the 3 A's (i.e., anxiety, agitation, and increased analgesic requirements) in the diagnosis of ACS in children are well established, some patients may experience only minimal pain. This challenges the correct and timely diagnosis of ACS in children, particularly if they are very young.

## 1 Introduction

Acute compartment syndrome (ACS) is a well-known and feared complication following traumatic injuries. When left untreated, the syndrome can be associated with severe limb-threatening consequences. ACS was first described in 1881 by Richard von Volkmann^[1]^ and is characterized by elevated pressure within a confined fascial space.^[2]^ The elevated pressure compromises blood circulation, leading to ischemia and finally to tissue necrosis. The ultimate consequence is loss of function of the involved extremity.

Excessive pain after an injury is the earliest and most reliable indicator of compartment syndrome^[3,4]^ and is present in 90%^[2,5]^ of patients with confirmed ACS. The “5 P's”, that is, pain, pallor, paresthesia, paralysis, and pulselessness, are the leading clinical symptoms^[6,1]^ in adults but these are not reliable predictors of ACS in young children. Instead, the “3 A's”, that is, anxiety, agitation, and increasing analgesic requirement, should be used as alternative signs of ACS in children.^[2,7,8]^

In the literature, “silent” compartment syndrome has been reported in a few cases. Silent compartment syndrome is defined as confirmed compartment syndrome without significant pain or absence of marked pain on passive motion.^[3,8]^ Here, we describe the development of silent compartment syndrome in a 2-year old patient with the aim to emphasize the challenges in diagnosing ACS in young children.

## 2 Case report

A 2-year old, previously healthy patient presented at the accident and emergency department of our institution after a heavy bistro table with a stone tabletop had fallen on the right hand. The patient presented in a good condition, and clinical examination showed soft but massive swelling of the hand and wrist region, modest pain, and inconspicuous neurovascular status with unrestricted function of fingers and wrist. No additional injuries were noted.

The parents of the patient provided written informed consent for publication of the case.

### 2.1 Investigations

**Figure 1:**
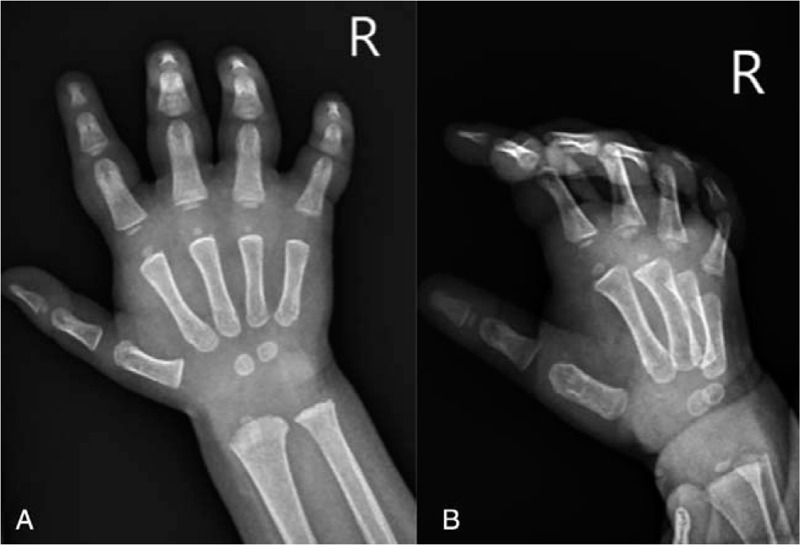
(A) Radiographic AP-image, (B) radiographic lateral-image.

Plain hand X-ray images showed no signs of fracture (Fig. 1). Due to the massive swelling of the hand and wrist, the patient was admitted to our in-patient ward to monitor the potential occurrence of compartment syndrome. The patient slept well during the night. The care records only mentioned massive swelling of the right hand.

**Figure 2:**
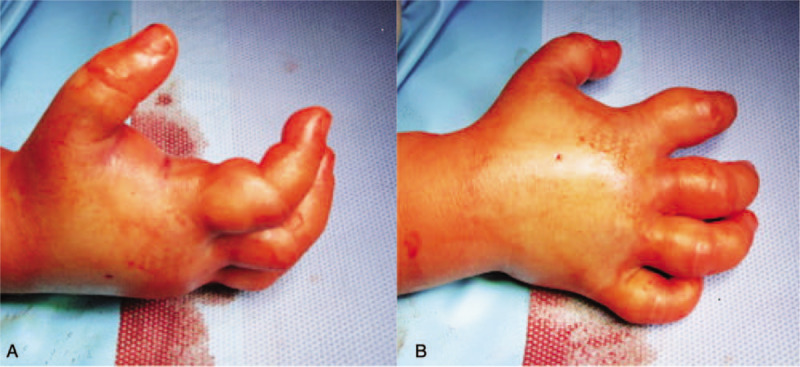
(A) Intrinsic minus position of the right hand, (B) central paleness on back of the hand.

At clinical re-assessment in the morning of the next day, we noted the patient's anxious discomfort, but the patient did still not complain of pain. In addition to the massive swelling of the hand, there was central paleness on both the palmar and dorsal aspects of the hand. The patient complained of paresthesia of the palm and dorsum of the right hand and held the hand in an intrinsic minus position (Fig. 2).

### 2.2 Differential diagnosis

Differential diagnoses of ACS of the hand comprise allergic reactions and swelling, edema, venous thrombosis, hematoma, thermal injuries, and fractures.

### 2.3 Treatment

#### 2.3.1 Pain treatment

The patient received a single, weight-adapted oral dose of ibuprofen on hospital admission. This resulted in adequate pain control.

#### 2.3.2 Surgical technique

**Figure 3:**
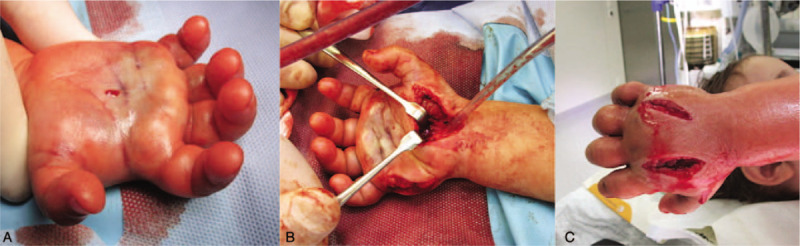
(A) Intrinsic minus position and central paleness of the right hand, (B) intraoperative situation; release of the transverse ligament of the carpal tunnel, (C) intraoperative situation; dorsal skin incisions.

Due to the dense swelling of the hand and impaired perfusion of the skin, the patient was taken to the operating room. Compartment pressure measured with a Stryker intra-compartmental pressure monitor system (Stryker Instruments, Kalamazoo, MI) under general anesthesia amounted to 60 mm Hg. Immediate fasciotomy of 10 hand compartments including thenar, hypothenar, adductor, and interossei compartments was performed as recommended in the literature.^[9]^ Additionally, we released the transverse ligament of the carpal tunnel. There was no muscle necrosis, and soft tissue re-perfusion was good (Fig. 3).

**Figure 4:**
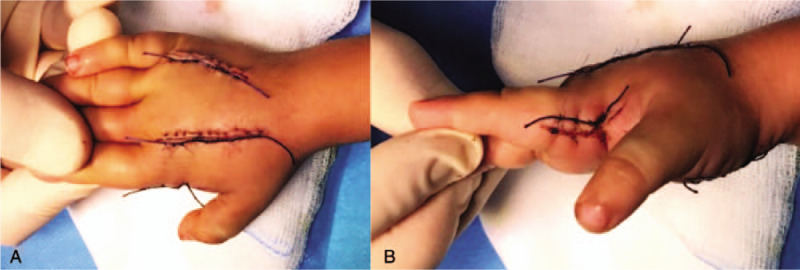
(A) Wound closure completed dorsal, day 7 after fasciotomy, (B) wound closure completed lateral, day 7 after fasciotomy.

The incisions were covered with Epigard synthetic skin substitute (Biovision GmbH, Ilmenau, Germany). The postoperative course was uneventful. Wounds were closed in 2 steps, with partial closure of the skin incisions on day 2 and final closure on day 7 after fasciotomy (Fig. 4).

#### 2.3.3 Outcome and follow-up

**Figure 5:**
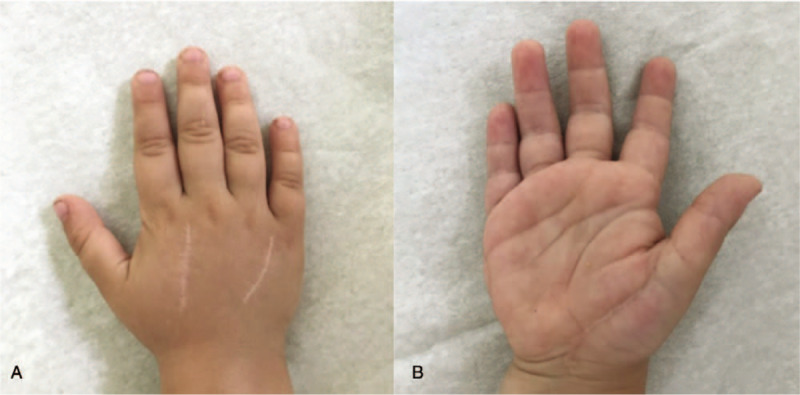
(A) Follow-up 1 year after fasciotomy, soft flexible scars, dorsal aspect, (B) follow-up 1 year after fasciotomy, soft flexible scars, volar aspect.

**Figure 6:**
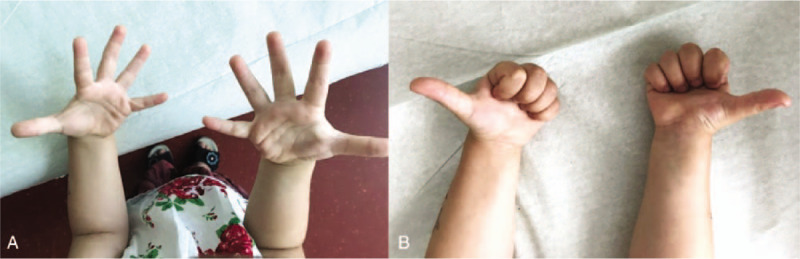
(A) Follow-up 1 year after fasciotomy, full range of hand motion achieved, dorsal aspect, (B) follow-up 1 year after fasciotomy, full range of hand motion achieved, volar aspect.

The patient was regularly followed up at our outpatient clinic. One year after injury, the patient showed unrestricted hand and wrist functions, and the parents reported that the patient was using the hand without pain during daily activities (Figs. 5 and 6).

## 3 Discussion

ACS in children is frequently associated with high-energy trauma^[2]^ such as motor vehicle collisions, falls from a height, crush injuries, and high-impact sports injuries. Typically, ACS is combined with fractures,^[4]^ especially of the lower extremities.^[7]^ Other causes can be postoperative swelling or bleeding, paravasates of intravenous drugs, tight casts, retained tourniquets, sepsis, and burns.^[2,10]^ In a computerized search of the National Pediatric Trauma Registry for all patients who had experienced a compartment syndrome during a 51-month period, Grottkau et al^[5]^ identified 132 cases of ACS aged less than 19 years. In this study, 60.3% of patients with suspected ACS were immediately fasciotomized. About 27.5% of patients were monitored in the in-patient ward for clinical signs indicative of ACS. A total of 10.7% of patients were referred to an intensive care unit. Development of ACS can be delayed as tissue pressures rise after variable times after injury. Royle et al^[11]^ described an average of 41 hours after initial injury until ACS becomes obvious. Thus, in-patient ward monitoring for at least 48 hours is recommended.

### 3.1 Diagnostic aspects of ACS in children

Presently, no laboratory tests or imaging studies for the timely diagnosis of ACS are available. Therefore, we did not undertake any laboratory tests in our patient. Pulse oximetry is an unreliable tool to diagnose the onset of ACS. Thus, early diagnosis of ACS relies on clinical findings, such as the presence of the 3 A's in children or 5 P's in adults. In our patient, we diagnosed ACS exclusively by clinical criteria and measured intra-compartmental pressure under anesthesia. Intra-compartmental pressure amounted to 60 mm Hg, confirming our clinical findings. Measurement of intra-compartmental pressure is invasive and painful and thus requires adequate anesthesia.

Our case showed that the absence of pain does not exclude ACS development. In the literature, we found only 2 reports including a total of 9 children and young adults who sustained ACS without severe pain. Lee et al^[8]^ described 5 children with a median age of 7 years “without the presence of significant pain at rest or on passive range of motion (PROM)”. Badhe et al^[3]^ described the development of ACS without significant pain in 4 neurologically normal young male patients. The authors concluded that pain is not a reliable clinical indicator for early ACS. Instead, paralysis, swelling, paresthesia, firm but compressible compartments, and increased requirement of pain medication were reliable early clinical symptoms.^[3,8]^ Our patient experienced a silent compartment syndrome, meeting one of the 3 A's (i.e., anxiety) and 3 of the 5 P's (i.e., pallor, paresthesia, and paralysis).

In general, the therapy of ACS is fasciotomy. The indication for fasciotomy is based on clinical findings and confirmed elevation of intra-compartmental pressure. Reliable and widely accepted indications for fasciotomy are available for adults only.^[7]^ A difference between diastolic blood pressure and compartment pressure (delta pressure) of 30 mm Hg or less is used as the threshold for fasciotomy. To the best of our knowledge, no generally accepted recommendations for the diagnosis of ACS in children are available. There are variable thresholds of compartment pressure (ranging from 20 to 40 mm Hg) serving as the recommended indication for fasciotomy. Some authors evaluated the physiologic compartment pressure in children and found them to be significantly higher than those in adults.^[12,13]^ The authors concluded that children seem to tolerate higher absolute compartment pressures and lower pressure gradients before ACS occurs.^[13]^ Unfortunately, the available data are still scarce, and no absolute value can be defined as the clear-cut indication for fasciotomy in children.^[13]^

### 3.2 Study limitations and strengths

The limitation of this case report is that we describe the successful therapy of a silent compartment syndrome in only 1 patient, and therefore our findings and conclusions cannot be generalized. Further case series are needed to establish valuable treatment guidelines.

The strengths of this report include the description of the typical aspects and challenges of a silent ACS in a young child. Our experience with the treatment of a silent ACS augments the scarce literature available and thus helps to improve the therapeutic management of affected children. In addition, this case may help to reduce severe medical consequences thus preventing more costly hospitalizations.

### 3.3 Conclusion

Our case illustrates the need to consider the possibility of a silent ACS in children. Although pain is commonly the leading symptom in ACS, the absence of moderate or severe pain in neurologically responsive patients does not necessarily exclude ACS. Moreover, because ACS can develop with some delay, a high index of clinical suspicion and frequently repeated clinical examinations are recommended for every patient, even those being monitored in the in-patient ward. In the absence of specific guidelines for children, we recommend to comply with the accepted threshold values for fasciotomy in adults.

In conclusion, surgeons and emergency physicians should be aware of the possibility of silent ACS to allow for timely diagnosis and surgical treatment of ACS, thus preventing long-term disability. We emphasize that surgeons should never be afraid of creating a large wound in suspected ACS; they should rather be concerned about tissue necrosis and long-term impairment of extremity function.

## Author contributions

**Conceptualization**: Benjamin Frei, Johannes Mayr.

**Data curation**: Benjamin Frei.

**Investigation:** Benjamin Frei.

**Methodology:** Johannes Mayr.

**Project administration:** Benjamin Frei, Vivienne Sommer-Joergenson.

**Supervision:** Stefan Holland-Cunz.

**Validation:** Johannes Mayr.

**Visualization:** Benjamin Frei, Vivienne Sommer-Joergenson.

**Writing – original draft:** Benjamin Frei.

**Writing – review & editing:** Johannes Mayr.
